# Ultrasonographic findings of re-epithelialized skin after partial-thickness burns

**DOI:** 10.1186/s41038-018-0122-3

**Published:** 2018-08-06

**Authors:** Jong Dae Kim, Suk Joon Oh, Sun Gyu Kim, Song Vogue Ahn, Yu Jin Jang, Ban Seok Yang, Ji Yun Jeong, Kwang Jo Kim

**Affiliations:** 1 Department of Burn Reconstructive Surgery, Bestian Seoul Hospital, Dogok-ro 429, Gangnam-gu, Seoul, Republic of Korea; 2 Department of Health Convergence, Ewha Womans University, Ewhayeodae-gil 52, Seodaemun-gu, Seoul, Republic of Korea; 3 Department of Radiology, Bestian Seoul Hospital, Dogok-ro 429, Gangnam-gu, Seoul, Republic of Korea; 4 Department of General Surgery, Bestian Seoul Hospital, Seoul, Republic of Korea

**Keywords:** Partial-thickness burn, Ultrasonography, Low-echogenic band, Healing time, Scar, Re-epithelialized skin

## Abstract

**Background:**

This study aimed to investigate the difference between ultrasonographic findings of normal skin and those of re-epithelialized skin after partial-thickness burns and to evaluate the relationship between these findings and clinical outcomes.

**Methods:**

This study retrospectively analysed the ultrasound images of re-epithelialized skin after partial-thickness burns and contralateral normal skin from January 2016 to December 2016. A total of 155 lesions from 148 patients were analysed with ultrasound images, and healing time was documented. The scar status of each lesion was evaluated through medical records and photographs. We analysed the difference in ultrasonographic findings between normal skin and re-epithelialized skin after partial-thickness burns and statistically analysed the relationship between healing time, scar status and ultrasonographic findings.

**Results:**

The re-epithelialized skin after partial-thickness burns was significantly thicker than the contralateral normal skin, and the echogenicity was significantly lower. The ultrasound images of the re-epithelialized skin after partial-thickness burns showed the characteristic findings of low-echogenic bands (LEB), and the proportion of LEB thickness is strongly correlated with healing time. In the multivariate analysis of scar status, only the proportion of LEB thickness was statistically significant.

**Conclusion:**

In this study, we found that there were ultrasonographic differences between re-epithelialized skin after partial-thickness burns and normal skin and that an LEB of varying thickness was formed after re-epithelialization. The thickness of the LEB in re-epithelialized skin after partial-thickness burns increased with healing time and was related to scar status.

## Background

Burn injuries have varied prognoses, ranging from scar-less wound healing to severe scar formation with or without contracture, depending on the depth of the wound. Because pre-existing burn scars are difficult to treat, it is important to predict and prevent severe scar formation to avoid functional and cosmetic losses.

In general, a useful method for predicting the degree of scarring is to assess the healing time of the wound [1–3]. However, the baseline healing time (re-epithelialization time) is physician-dependent, and the healing time error increases for patients who do not receive daily dressing changes (especially outpatients). Therefore, a more accurate prediction of scarring requires more information about the colour, texture, and pliability of wounds in addition to healing time.

Traditionally, scar evaluation has relied on physical examination based on an expert experience; however, more recently, a variety of objective assessment tools have been developed for scar evaluation [4, 5]. In objective assessments, ultrasound imaging is an effective modality for measuring skin and scar thickness [5–7]. Ultrasonography provides objective and quantitative information with which to identify the current state of the scar and to evaluate treatment results [8–10]. In ultrasonographic wound evaluation, Dunkin et al. [11] reported that wounds were represented as an area of low echogenicity in the dermis and that the echogenicity of the wound changed as healing progressed. In their human wound model, deeper wounds were expressed in deeper low-echogenic areas on ultrasound images.

As part of a series of objective scar evaluations, our burn centre performed early ultrasound examinations of burn wounds after re-epithelization was complete. We identified significant differences in the ultrasonographic findings between re-epithelialized skin after partial-thickness burns and normal skin.

The current study was a pilot feasibility study to investigate the difference between the ultrasonographic findings of normal skin and re-epithelialized skin after partial-thickness burns and to evaluate the relationship between these findings and clinical outcomes.

## Methods

### Study design

This study retrospectively analysed the ultrasound images of re-epithelialized skin after partial-thickness burns and contralateral normal skin from January 2016 to December 2016.

All enrolled burn wounds were conservatively treated without surgical procedures following the standard burn treatment protocol of our centre. Sites with a known re-epithelialization time based on medical records were selected as regions of interest (ROIs). All ROIs achieved re-epithelialization between 7 and 28 days and were evaluated by ultrasound within 14 days after re-epithelialization.

Burn patients older than 70 years who had systemic disease or who had total body surface area (TBSA) burns > 10% were excluded from this study. Lesions with eczema, dermatitis or hyperkeratosis at the time of ultrasound examination were also excluded.

All lesions were treated with conservative therapies, including moisturizers and scar ointments, after re-epithelialization. However, special therapies, such as pressure garments and scar massage, were not applied before ultrasound examination.

Ultrasound examinations, analysis of ultrasound images and evaluation of clinical outcomes were performed by the authors.

Ethical approval was obtained by the institutional review board of our hospital.

### Ultrasound evaluation

Before ultrasound examination, the ROIs in each lesion were determined as the areas with the clear and longest healing times by reviewing the medical records and photographic records. The contralateral area of each ROI was defined as the control normal skin, and ultrasound examination was performed during the same examination session.

An Ultrascan UC22 (Courage + Khazaka electronic Gmbh, Cologne, Germany) was used in this study. The central frequency was 22 MHz, the focus length was 11 mm, the scanning width (lateral) was 12.8 mm, the lateral resolution was 33 μm, the axial resolution was 72 μm, and the digitalization depth was 8 mm. After obtaining ultrasound images, thickness and echogenicity were evaluated on a personal computer using the software provided by the manufacturer of the ultrasound device.

**Fig. 1:**
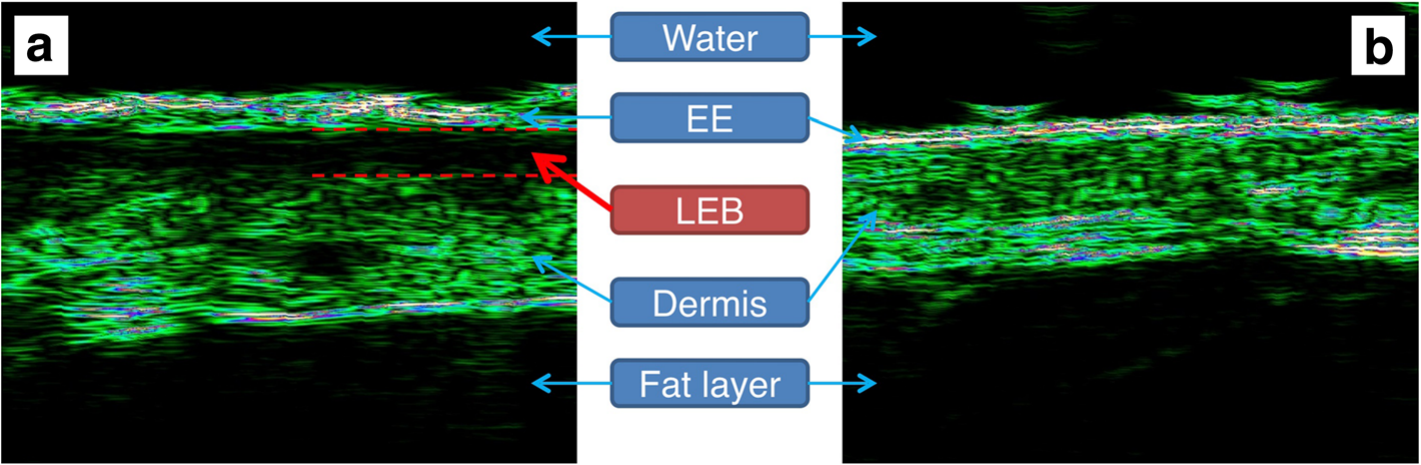
Ultrasonographic findings of the skin. **a** Re-epithelialized skin after a partial-thickness burn (thigh, healing time 19 days). **b** Contralateral normal skin. *EE* entrance echo, *LEB* low-echogenic band

In the ultrasound images of the ROIs, the entrance echo (EE), low-echogenic band (LEB) and remaining dermis were manually determined according to their echogenicity. Skin was defined as starting from the beginning of the EE to the dermal-fat interface. The low-echogenic areas produced by burn injury were ultrasonographically similar to the subepidermal LEB of photoaging skin [12, 13]; thus, we also used the term LEB. LEBs were defined from the endpoint of the EE to the point where echogenicity began to increase significantly. The remaining dermis of the ROI was defined as the area excluding the EE and LEB in the skin. LEBs were recognized by the relatively low echogenicity between the EE and the remaining dermis; both boundaries of the LEB were clear (Fig. 1). One observer had set LEB in each image to be a space between the EE and remaining dermis with a distinctly low echogenicity. The distinction between EE, LEB and residual dermis was based on the judgement of the observer, and no clinical information was provided to the observer when determining the LEB.

In the ROI, the skin thickness, LEB thickness and remaining dermal echogenicity were measured. In the contralateral normal skin, the skin thickness and dermal echogenicity were measured. In each observation, the ratio of skin thickness was calculated as the ROI value/control value, and the proportion of LEB thickness was calculated as LEB thickness/skin thickness of the ROI. The thickness was measured directly in millimetres on the image using the software provided by the manufacturer of the ultrasound device. Each value represents the average of three measurements of the thickness at the centre of the image. The echogenicity was automatically calculated by the software in a manually set rectangular space. In addition, each value represents the average of three measurements.

### Clinical outcome measurement

The healing time of all ROIs was defined as the number of days required for re-epithelization. A total of 91 ROIs had been observed to assess scar status. The scar status of each lesion was classified as normotrophic or hypertrophic by retrospectively assessing the condition at 3 months after re-epithelization using photographs and medical records.

### Data analysis

Paired *T* tests were used to compare the ultrasonographic findings of re-epithelialized skin and normal skin and to compare the findings according to scar status. Pearson’s correlation analysis was used to determine the relationship between ultrasound findings and wound-healing time. Univariate and multivariate logistic regression analyses were used to examine the association between the ultrasonographic findings and scar status. *p* < 0.05 was considered statistically significant. All statistical analyses were performed using R Statistical Software (version 3.4.0; R Foundation for Statistical Computing, Vienna, Austria).

## Results

### Clinical characteristics of ROIs

**Fig. 2:**
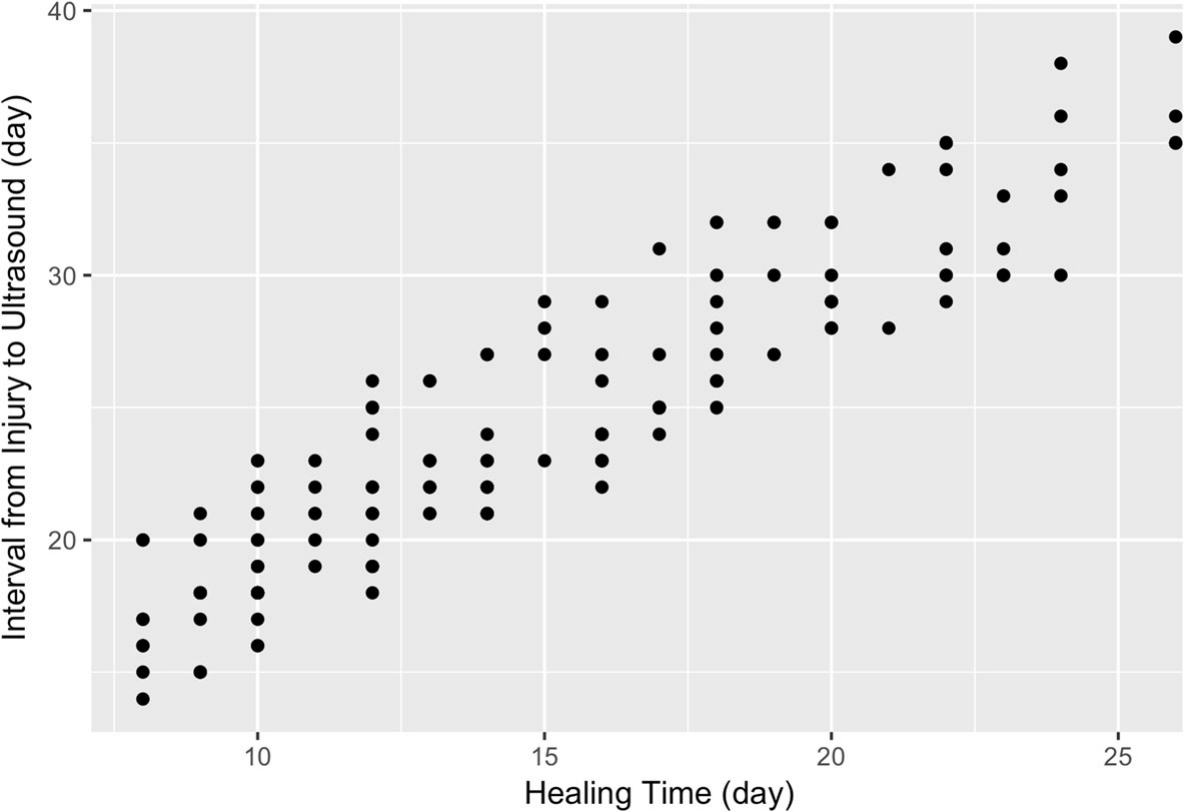
Time interval of the burn patients from injury date to ultrasound evaluation date. The mean was 24.28 days, and the standard deviation was 5.52 days

A total of 155 lesions from 148 patients and their contralateral normal skin were analysed (Table 1). The mean value of healing time was 14.88 days (SD = 4.84). The mean time interval from the injury date to the ultrasound evaluation date was 24.28 days (SD = 5.52) (Fig. 2). The mean value of the time interval from the re-epithelialization date to the ultrasound evaluation date was 9.40 days (SD = 2.22).

**Table 1:** Clinical characteristics of regions of interest of the burn patients

Variable		*N*
Sex		
	Female	85
	Male	70
Age (year)	Mean (SD)	21.79 (16.10)
Healing time (day)	Mean (SD)	14.88 (4.84)
Evaluation interval^a^ (day)	Mean (SD)	9.40 (2.22)
Site		
	Arm	27
	Leg	75
	Hand/Foot	19
	Trunk	10
	Joint	24
Cause		
	Scald	97
	Contact	48
	Flame	7
	Etc.^b^	3
Scar status		
	Normotrophic	74
	Hypertrophic	17
	Undetermined	64

^a^Time interval from the re-epithelialization date to the ultrasound evaluation date

^b^Electric arc, steam, and friction

*SD* standard deviation

### The difference between the ultrasonographic findings of normal skin and re-epithelialized skin after partial-thickness burns

**Fig. 3:**
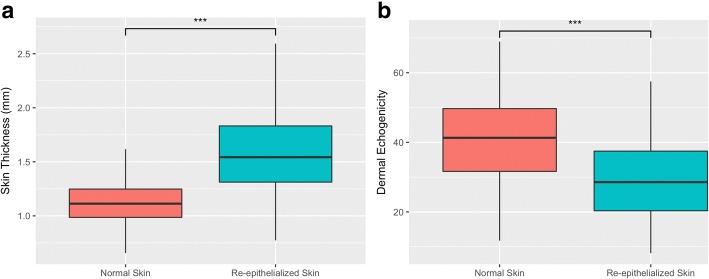
Differences in ultrasonographic findings between re-epithelialized skin after a partial-thickness burn and contralateral normal skin. **a** Difference of skin thickness. **b** Difference of dermal echogenicity. *p< 0.05, **p < 0.01, ***p < 0.001, ns = *p* > 0.05

On ultrasound images, re-epithelialized skin was significantly thicker than contralateral normal skin. The mean value of normal skin thickness was 1.136 mm (SD = 0.248), and the mean value of re-epithelialized skin after partial-thickness burns thickness was 1.567 mm (SD = 0.357; *p* < 0.001) (Fig. 3a).

Ultrasonographic echogenicity was also significantly different between normal dermis and the remaining dermis of re-epithelialized skin after partial-thickness burns. The mean dermal echogenicity value of normal skin (without EE) was 40 (SD = 11.5), and the mean value of the remaining dermal echogenicity (below the low-echogenic area) was 29 (SD = 11.7; *p* < 0.001) (Fig. 3b).

**Fig. 4:**
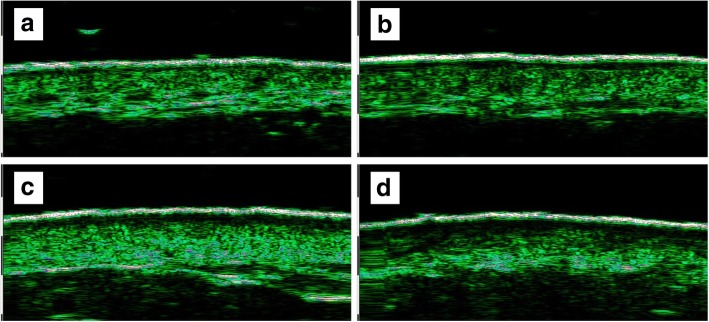
Ultrasound image of re-epithelialized skin after partial-thickness burns according to healing time (re-epithelialization time). **a** Healing time was 11 days, and the proportion of low-echogenic band (LEB) thickness was 14%. **b** Healing time was 16 days, and the proportion of LEB thickness was 20%. **c** Healing time was 19 days, and the proportion of LEB thickness was 25%. **d** Healing time was 26 days, and the proportion of LEB thickness was 30%

Ultrasound images of re-epithelialized skin after partial-thickness burns showed obvious low-echogenic areas under the EE. This finding was found in almost all lesions and was easily distinguished even in superficial lesions with less than 10 days of healing time. These low echoic areas seemed morphologically similar to the subepidermal LEBs observed on ultrasound examination of photoaging skin (Fig. 4).

### Correlation between the proportion of LEB thickness and healing time

**Fig. 5:**
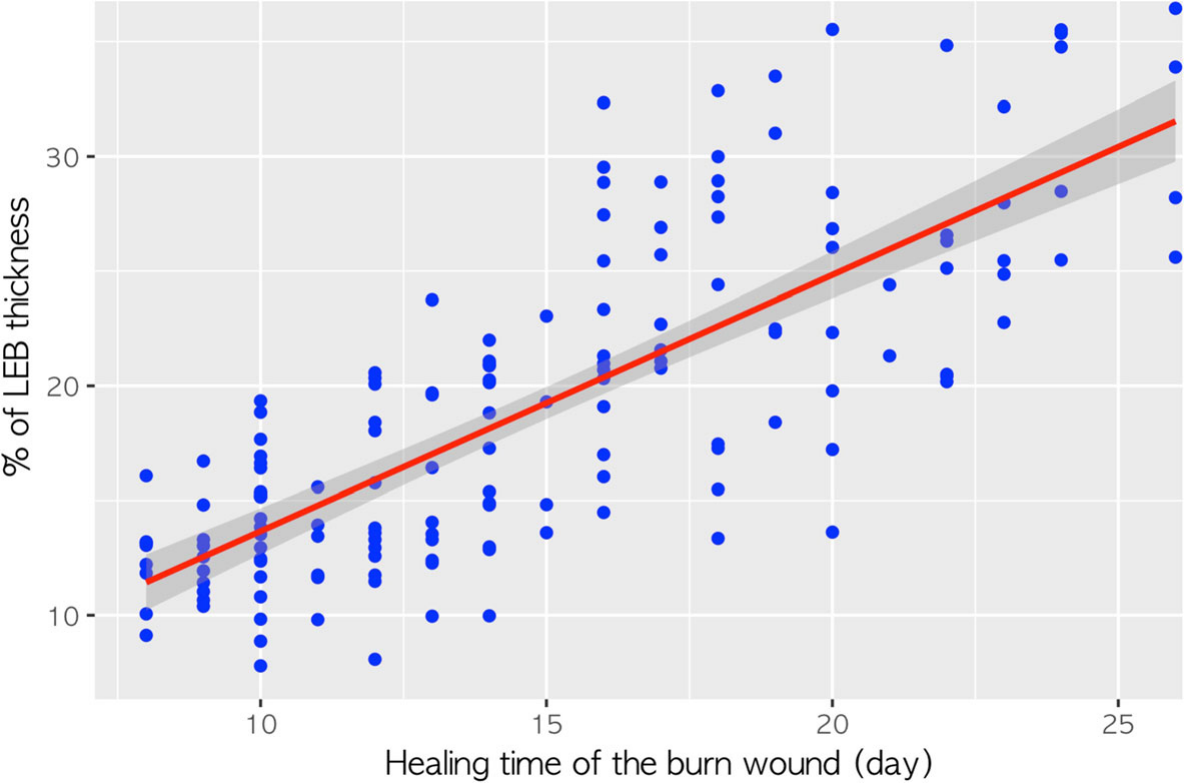
Scatter plot of low-echogenic band (LEB) thickness and healing time of re-epithelialized skin after partial-thickness burns (Pearson’s correlation coefficient = 0.775, *p* < 0.001)

The scatter plot in Fig. 5 illustrates a linear relationship between the proportions of LEB thickness and healing time. There was a strong correlation between the proportion of LEB thickness and healing time (Pearson’s correlation coefficient = 0.775, *p* < 0.001). The time interval from injury to ultrasound was also strongly correlated with the proportion of LEB thickness (Pearson’s correlation coefficient = 0.714, *p* < 0.001).

### Ultrasonographic findings associated with scar status

The mean value of the proportion of LEB thickness was 17.40% (SD=5.41) in the normotrophic group and 29.97% (SD=4.30) in the hypertrophic group. The difference in the proportion of LEB between the two groups was statistically significant (*p* < 0.001).

**Fig. 6:**
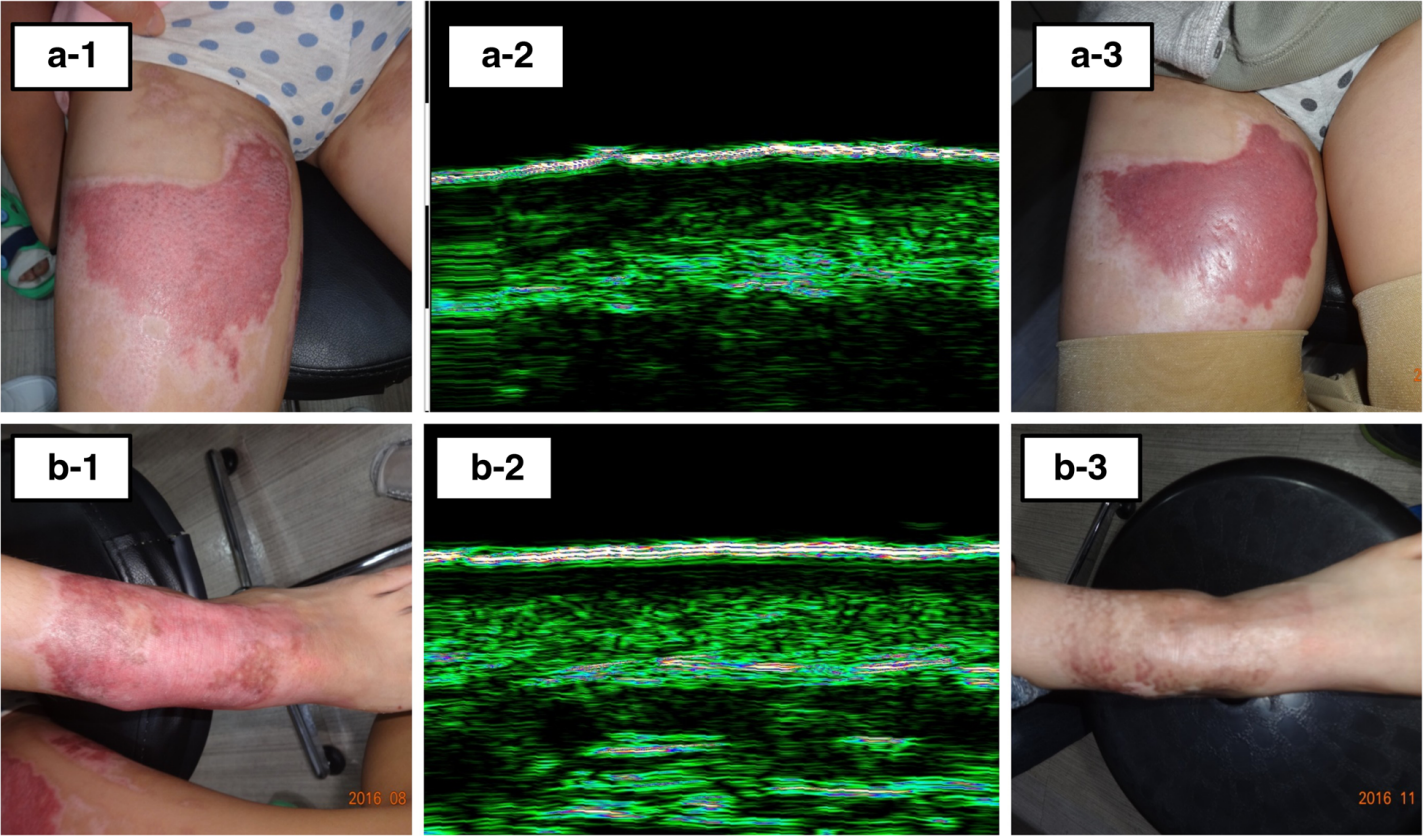
Clinical outcomes and initial ultrasound images of the scald burn in the same patient. The healing time of the right thigh was 24 days (**a-1**). The proportion of low-echogenic band (LEB) thickness was 35% (**a-2**), and a hypertrophic scar developed after 3 months (**a-3**). The healing time of the left ankle was 23 days (**b-1**). The proportion of LEB thickness was 25% (**b-2**), and normotrophic scar status was maintained (**b-3**)

In the multivariate analysis of statistically significant variables in the univariate analysis, only the proportion of LEB thickness was statistically significant (regression coefficient = 0.574, odds ratio = 1.775, *p* = 0.019) (Table 2). A typical scar status at 3 months after re-epithelialization and the initial ultrasound images are shown in Fig. 6.

**Table 2:** Logistic regression analysis of ultrasound findings associated with scar status

Ultrasound Finding	Mean(SD)		Univariate			Multivariate		
	NS (*n* = 74)	HS (*n* = 17)	B	OR	*P* value	B	OR	*P* value
Skin thickness of re-epithelialized skin (mm)	1.510 (0.318)	1.658 (0.345)	0.001	1.001	0.097			
EE thickness of re-epithelialized skin (mm)	0.126 (0.014)	0.127 (0.018)	0.008	1.009	0.646			
Remained dermal echogenicity of re-epithelialized skin	30.653 (10.863)	20.984 (10.598)	− 0.092	0.912	0.003**	− 0.047	0.039	0.445
LEB thickness of re-epithelialized skin (mm)	0.241 (0.098)	0.453 (0.102)	0.019	1.019	< 0.001***	0.005	1.005	0.533
Skin thickness ratio^a^	1.357 (0.262)	1.521 (0.438)	1.560	4.756	0.111			
EE thickness ratio^a^	1.023 (0.160)	1.006 (0.155)	− 0.686	0.503	0.752			
Dermal echogenicity ratio^a^	0.783 (0.262)	0.612 (0.294)	− 2.920	0.054	0.066			
Proportion of LEB thickness (%)^b^	17.398 (5.410)	29.971 (4.305)	0.575	1.776	< 0.001***	0.574	1.775	0.019*
Healing time (days)	14.068 (4.473)	21.294 (3.405)	0.349	1.417	< 0.001***	0.051	1.052	0.838
Interval from injury to ultrasound (days)	23.541 (4.963)	31.059 (4.67)	0.275	1.317	< 0.001***	− 0.019	0.037	0.918

*SD* standard deviation, *B* regression coefficiency, *OR* odd ratio, *NS* normotrophic scar, *HS* hypertrophic scar, *EE* entrance echo, *LEB* low-echogenic band

^a^Ratio of re-epithelialized skin to contralateral normal skin

^b^Percentage of LEB thickness in re-epithelialized skin thickness

*P < 0.05, **P < 0.01, ***P < 0.001

## Discussion

In this study, we found that re-epithelialized skin after partial-thickness burns was thicker and had lower ultrasonographic echogenicity than normal skin and that an LEB was present, as in photoaging skin. We also found that the thickness of the LEB was closely correlated to healing time and was associated with the type of scar.

The water content of the dermis is principally regulated by the pressure of the interstitial fluid, which, in turn, is determined by the distensibility of the collagen network and the water-holding capacity of glycosaminoglycans [12, 14]. Comparative studies of ultrasonography and nuclear magnetic resonance spectroscopy showed that dermal water content can be directly correlated with dermal echogenicity [15]. Greater fluid content in tissue results in a decrease in echogenicity readily detectable by ultrasound [16]. In acute burn wounds, vasoactive mediators, such as prostaglandins, histamine, and bradykinin, can lead to oedema by altering endothelial cell and basement membrane function to increase permeability [17, 18]. In this study, we found that dermal echogenicity was decreased and that skin thickness was increased in re-epithelialized skin after partial-thickness burns compared to those in contralateral normal skin, which is thought to be the effect of oedema. This difference tended to be prominent when the healing time was long and decreased with wound maturation over time (data not shown).

In the wound-healing process, granulation tissue, comprising procollagen, elastin, proteoglycans, and hyaluronic acid, allows the ingrowth of new blood vessels [19, 20]. The extracellular matrix is immature, with fine bundles of collagen in a predominantly linear (horizontal) organization and an increased number of blood vessels [21]. Therefore, newly organized tissue during the healing process is highly hydrated and shows very low echogenicity on ultrasound images.

Hoffmann et al. [22] described the poor internal echoes in lesions after cryosurgery at the end of the healing process as associated with newly formed fine-fibrillary connective tissue (granulation tissue). Rippon et al. [23] analysed ultrasound scans of wounds and showed that early granulation tissue was echo-poor because of the low echogenicity of cellular infiltrates. The researchers also concluded that ultrasound can be used to visualize and quantify fibrous granulation tissue accumulation in wounds. In a human wound model, Dunkin et al. [11] demonstrated that the wound was represented as an area of echolucency in the dermis. In their study, the thickness of the low-echogenic area produced by wounds varied depending on the injury depth.

In the present study, we also found a low-echogenic area in the re-epithelialized skin after partial-thickness burns. Because most burn injuries cause surface damage, this low-echogenic area takes the form of a ‘band’ similar to the ‘subepidermal LEB’ observed in the ultrasound of photoaging skin [12–14].

This ultrasonographic LEB is found in all lesions, even in wounds with a healing time of fewer than 10 days. This band was thicker and more significant with longer healing time. Because each patient and each body site have variable skin thickness, we used the proportion of LEB thickness among the total skin thickness to assess the correlation with healing time, and a strong correlation was observed. is influenced not only by the depth of the wound but also by various factors; however, when the wound is deeper, the healing time becomes longer and the newly organized tissue becomes thicker, which can be observed in the form of an LEB through ultrasound. Based on these results, we suggest that ultrasound can be used to retrospectively estimate the depth wounds with uncertain clinical information.

Wound tissues change over time during the wound-healing process, so timing of the ultrasound evaluation may affect the result. Usually, hypertrophic changes begin 1 to 2 months after re-epithelialization. To assess the condition before hypertrophic change, we selected ROIs evaluated before 14 days after re-epithelialization. The time interval from the injury date to ultrasound was the sum of the healing time and the time interval from re-epithelialized date to ultrasound. Because a deviation in the time interval from the re-epithelialized date had relatively low deviation, the time interval from injury date was more affected by the healing time. Therefore, the proportion of LEB thickness was inevitably correlated to the time interval from the injury date as well as the healing time. In addition, the time interval from the injury date was statistically significant only in the univariate analysis, not in the multivariate analysis.

In human burns, delayed wound healing is a significant risk factor for hypertrophic scarring [1], and healing time is an important factor in the prediction of scarring and the selection of scar prevention methods [2, 3]. Because of the strong relationship between the proportion of LEB thickness and healing time, we hypothesize that the proportion of LEB thickness is also a useful prognostic factor in the prediction of scarring. In the univariate logistic regression analysis, both the healing time and the proportion of LEB thickness were statistically significant factors affecting scar status. The proportion of LEB thickness was the only statistically significant factor in the multivariate model (Table 2). These results indicate that the proportion of LEB thickness of post-burn skin may have a prognostic value for scarring similar to that of healing time.

In this study, the LEB thickness was closely related to the healing time associated with wound depth. The LEB thickness is expected to be related to the wound depth. Thin LEB lesions without hypertrophy may be limited to papillary dermal injury, and thick LEB lesions with hypertrophy may include reticular dermal injury. However, 22 MHz ultrasound cannot distinguish between the papillary dermis and the reticular dermis. Ultrasonography with a higher MHz is required for more detailed inspection.

The ultrasound examination, which is reproducible and valid, results in no damage to the wound and allows serial scans to be obtained, allowing temporal changes within the wound to be monitored [8]. In objective scar assessment, the ultrasound technique is an effective modality for measuring scar thickness [5]. Ultrasonography provides objective and quantitative information with which to identify the current state of the scar and to evaluate treatment results [9]. Ultrasound examination of early re-epithelialized skin after partial-thickness burns is valuable not only for the estimation of scarring but also for the serial objective scar assessment to observe the progress of the scar.

Clinical practice to prevent hypertrophic scars requires great effort and cost. For effective clinical practice, it is necessary to select the wound to which such efforts and costs apply. Early ultrasound evaluation of re-epithelialized skin after partial-thickness burns can inform the potential to form a hypertrophic scar and help to determine the management strategy. Re-epithelialized skin after partial-thickness burns with thick LEB in early ultrasound evaluation may be associated with a longer healing time and higher scar potential and need a strict scar prevention strategy. In a human model study, Dunkin et al. [11] reported that the mean threshold depth to leave a scar on the lateral aspect of the hip was 33.1% of total skin thickness. In our data, the mean value of the proportion of LEB thickness in the hypertrophic scar group was 29.97% (SD=4.30). In our centre, a strict scar management protocol is recommended for re-epithelialized skin after partial-thickness burns with a proportion of LEB thickness above 25%.

The proposed ultrasound device operates at a frequency of 22 MHz. Agabalyan et al. [24] showed that a 20 MHz probe could not provide sufficient penetration and information to evaluate scar thickness. These authors recommended using a transducer set between 5 and 10 MHz for measuring deeper dermal thickness. In our experience, the penetration thickness of the 22 MHz probe that we used was limited to 3 mm although the manufacturer described between 6 and 8 mm. In this study, however, our ROI was the recently re-epithelialized skin; therefore, it was sufficient to use the 22 MHz probe rather than the hypertrophic scar.

The ultrasound examination was simple to set up and use and required little training to operate. The time taken to record a scan was very short. We also performed the ultrasound examinations on an outpatient basis. A typical scan took only approximately 1 min and caused no discomfort to the patient. These scans allow us to observe the status of re-epithelialized skin after a burn and determine the most cost-effective management plan for scar treatment. In addition, the scanning results provide a computerized two dimensional (2D) image that is intuitive and understandable to the patient in real time, which can be an effective way to explain the patient’s current condition and treatment plan.

This study is a retrospective analysis. Because the data on healing time were obtained through medical records and the ROI was set manually, it is possible that information bias exists. Another possible limitation is that the scar status was evaluated by dichotomy, and other factors, such as erythema and pliability, were not considered. There was no control for other factors (e.g., wound location, sex, or age) that could affect skin thickness and echogenicity or for other factors that could affect scar formation. Further studies are needed to evaluate the prognostic value of skin ultrasonography after re-epithelialization of burn injuries.

## Conclusions

In this study, we found that there were ultrasonographic differences between re-epithelialized skin after partial-thickness burns and normal skin and that an LEB of various thicknesses was formed after re-epithelialization. The thickness of the LEB in the re-epithelialized skin after partial-thickness burns increased with healing time and was related to scar status.

## Abbreviations

EE: Entrance echo
LEB: Low-echogenic band
ROI: Region of interest
SD: Standard deviation
TBSA: Total body surface area
